# Vitamin C Supplementation During Intensive Care Unit Stay Is Associated With Improved Outcomes in Critically Ill Patients With Sepsis‐Induced Coagulopathy: A Cohort Study

**DOI:** 10.1002/fsn3.71263

**Published:** 2025-11-24

**Authors:** Ji Li, Fulin Li, Dan Luo, Enling Liu, Haibing Lan

**Affiliations:** ^1^ Department of Intensive Care Unit The Second Affiliated Hospital of Nanchang University Nanchang Jiangxi P.R. China

**Keywords:** 28‐day hospital mortality, MIMIC‐IV database, propensity score matching, sepsis‐induced coagulopathy, vitamin C

## Abstract

This study aimed to investigate whether vitamin C provides survival benefit in critically ill patients with sepsis‐induced coagulopathy (SIC). Patients with SIC admitted to the ICU were identified from the Medical Information Mart for Intensive Care (MIMIC)‐IV database. The exposure factor was vitamin C supplementation during the ICU stay. The primary outcome was 28‐day all‐cause mortality, the secondary outcome was 60‐day all‐cause mortality, in‐ICU mortality, and the extended outcomes included duration of mechanical ventilation, ICU stay, and hospital stay. Both propensity score matching (PSM) and multivariable Cox models were employed to adjust for potential confounders. Sensitivity analyses and subgroup assessments were also performed to verify the study findings. A total of 18,283 eligible patients were enrolled in the entire unmatched cohort, and 2251 patients were included in the matched cohort. In PSM analysis, vitamin C supplementation was associated with decreased 28‐day all‐cause mortality (HR, 0.52; 95% CI, 0.40–0.69; *p* < 0.001), 60‐day all‐cause mortality (HR, 0.69; 95% CI, 0.54–0.86; *p* < 0.001), and ICU mortality (OR, 0.70; 95% CI, 0.50–0.97; *p* < 0.001). However, the duration of mechanical ventilation was significantly longer in the vitamin C group (median 42 vs. 53 h, *p* < 0.001). During ICU admission, continuous vitamin C supplementation for over 6 days was associated with improved 28‐day in‐hospital outcomes in patients with SIC. Sensitivity analysis using the unmatched cohort confirmed these findings (HR, 0.58; 95% CI, 0.45–0.75; *p* < 0.001). Vitamin C supplementation may reduce mortality in critically ill patients with SIC. However, further high‐quality prospective studies are still needed to validate these findings.

## Graphical abstract

Based on a large retrospective cohort of critically ill patients with sepsis‐induced coagulopathy, this study found that intravenous vitamin C supplementation during ICU stay was significantly associated with reduced 28‐ and 60‐day all‐cause mortality. The survival benefit was most pronounced when vitamin C was administered continuously for more than 6 days. However, vitamin C was also associated with prolonged durations of mechanical ventilation and ICU stay.

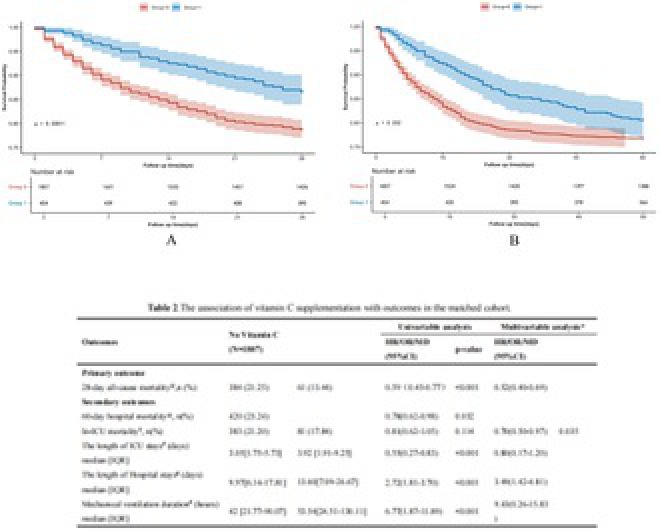

**Abbreviations**
AKI: acute kidney injury
APSIII: acute physiology score III
APTT: activated partial thromboplastin time
BUN: blood urea nitrogen
CHD: coronary heart disease
DBP: diastolic blood pressure
HR: heart rate
ICU: intensive care unit
INR: international normalized ratio
OASIS: oxford acute severity of illness score
pCO_2_
: partial pressure of carbon dioxide
PLT: platelet count
pO_2_
: partial pressure of oxygen
RDW: red blood cell width
RR: respiratory rate
SBP: systolic blood pressure
SIC: sepsis‐induced coagulopathy
WBC: white blood cell count

## 1 Introduction

According to 2019 statistics, nearly 50 million patients worldwide are affected by sepsis annually, with over 11 million sepsis‐related deaths—exceeding mortality from ischemic heart disease and cancer (Rudd et al. 2020). Sepsis often induces varying degrees of coagulation dysfunction, which may progress to overt disseminated intravascular coagulation (DIC) in severe cases (Iba and Levy 2019). The coexistence of sepsis and coagulopathy significantly increases mortality and represents a major clinical challenge. Sepsis‐induced coagulopathy (SIC), characterized by systemic activation of coagulation, is considered an early stage of DIC (Iba et al. 2019). Approximately 25% of septic patients develop SIC in the early phase, nearly a quarter of whom do not survive (Iba et al. 2024). In‐hospital mortality reaches 56.1% in patients with severe SIC (Lyons et al. 2018). Recognizing the critical impact of coagulation system dysfunction in sepsis, the International Society on Thrombosis and Haemostasis (ISTH) has established diagnostic criteria for SIC (Iba et al. 2020). A SIC score ≥ 4 is associated with mortality exceeding 30%, and this scoring system demonstrates superior predictive value for sepsis‐related mortality compared to conventional DIC criteria (Yamakawa et al. 2019).

Vitamin C is an essential micronutrient involved in regulating oxidative stress, inflammatory responses, and immune cell function, thereby conferring protective effects on multiple organ systems (Wang et al. 2023; Tyml 2011; Wilson 2009). Studies have shown that vitamin C deficiency is common in patients with sepsis and septic shock, often requiring high‐dose intravenous supplementation to restore plasma levels (Carr et al. 2017; Hwang et al. 2019; Long et al. 2003; de Grooth et al. 2018). However, the LOVIT trial reported that intravenous vitamin C administration was associated with a higher risk of death or persistent organ dysfunction at 28 days compared to placebo in septic patients receiving vasopressors (Lamontagne et al. 2022). A secondary analysis of the trial revealed that there was no difference in mortality between the vitamin C and placebo groups during the 4‐day vitamin C administration, but in the vitamin C group mortality greatly and briefly increased immediately after vitamin C was stopped, which is called the rebound effect in pharmacology (Hemilä and Chalker 2023, 2024).

Vitamin C has been shown to exert dual procoagulant and anticoagulant properties through multiple mechanisms (Kinnunen et al. 2017; Ranucci et al. 2013; Gielen et al. 2014). Early animal studies indicated that high‐dose vitamin C might promote thrombosis, while other studies reported its ability to ameliorate coagulation abnormalities and attenuate platelet decline in sepsis models (Fisher et al. 2012; Tyml 2017). Additionally, vitamin C can modulate the expression of various plasma proteins involved in inflammation and coagulation (Parahuleva et al. 2016; Tousoulis et al. 2003). Therefore, this study aims to explore the potential correlation between the use of vitamin C and the prognosis of patients with SIC through a large retrospective data set.

## 2 Materials and Methods

### 2.1 Study Design and Data Source

The data were obtained from the MIMIC‐IV dataset (version 3.1) (Johnson et al. 2023). The MIMIC‐IV database is a publicly available large‐scale database containing ICU patient data from Beth Israel Deaconess Medical Center in Boston, USA, widely used in medical research. It provides researchers with comprehensive clinical data including medical records, test results, and treatment documentation, encompassing over 90,000 ICU admissions from 2008 to 2022. The data were anonymized to protect patient privacy while maintaining integrity and quality. The Institutional Review Boards (IRBs) of Beth Israel Deaconess Medical Center and Massachusetts Institute of Technology approved this study. The authors completed the NIH online training course and passed the exam, obtaining access to extract data.

### 2.2 Study Population

This study selected patients who were diagnosed with SIC within 24 h of ICU admission. Sepsis was defined as life‐threatening organ dysfunction according to the Sepsis‐3 diagnostic criteria from the International Guidelines for Management of Sepsis and Septic Shock. SIC was diagnosed per the ISTH criteria, which integrate three key parameters: INR, platelet count, and SOFA score. The detailed criteria for the diagnosis of SIC can be found in Table S1. The exclusion criteria were as follows: (1) age < 18 years; (2) ICU length of stay < 24 h; (3) for patients with multiple ICU admissions, only the first admission was retained and analyzed.

### 2.3 Exposure and Outcomes

Medication exposure was determined through the prescription table. Vitamin C exposure was simply defined as the administration of vitamin C supplements during the ICU stay, without additional restrictions. The primary outcome was 28‐day all‐cause mortality. The secondary outcomes included 60‐day all‐cause mortality, in‐ICU mortality, length of ICU stay, length of hospital stay, and duration of mechanical ventilation.

### 2.4 Data Extraction

Data were extracted from the MIMIC‐IV (v3.1) database via the Structured Query Language (SQL) via Navicat Premium (version 17), following the platform's data access protocols. Demographic data, vital signs, comorbidities, laboratory tests, scoring systems, medication treatments, and survival data were extracted. Demographics included age and gender. Vital signs included heart rate (HR), systolic blood pressure (SBP), diastolic blood pressure (DBP), and respiratory rate (RR). The comorbidities included acute kidney injury (AKI), coronary atherosclerotic heart disease (CHD), hypertension, diabetes, liver cirrhosis, and cancer. The laboratory measurements included pH, partial pressure of oxygen (pO_2_), partial pressure of carbon dioxide (pCO_2_), anion gap, red cell distribution width (RDW), hemoglobin, white blood cell count (WBC), platelet count (PLT), lactate, alanine aminotransferase (ALT), total bilirubin (Tbil), serum creatinine, blood urea nitrogen (BUN), calcium, potassium, glucose, international normalized ratio (INR), and activated partial thromboplastin time (APTT). Treatments included vasopressor use, mechanical ventilation, heparin sodium and glucocorticoid use. The scoring systems included the acute physiology score III (APSIII), Oxford acute severity of illness score (OASIS), and SIC scores. Data collection was restricted to the first 24 h post‐ICU admission. For parameters with multiple measurements, the initial value was used.

### 2.5 Statistical Analysis

Variables with over 20% missing data were excluded. Multiple imputation was performed using the mice package in R to ensure data completeness and analytical validity (Allison 2000) (see Table S2). Multicollinearity was evaluated via variance inflation factors (VIF > 5, indicating severe collinearity). Variables exceeding this threshold (see Table S3) were sequentially excluded to optimize model stability. Mann–Whitney *U* test was used to analyze non‐normally distributed continuous variables, and the results were expressed as median and interquartile range. Chi‐square test was used to compare categorical variables between the two groups. For the primary outcome, the Cox proportional hazards model was employed to calculate the hazard ratio (HR) and 95% confidence interval (CI). The Kaplan–Meier method and the log‐rank test were used to estimate and compare the cumulative incidence of 28‐ and 60‐day all‐cause mortality. For dichotomous secondary outcomes, the logistic regression model was applied to calculate the odds ratio (OR) and 95% CI. For continuous secondary outcomes, the Hodges–Lehmann method was utilized to calculate the median difference (MD) and 95% CI. For all analyses, a two‐tailed *p* < 0.05 was deemed statistically significant. All the statistical analyses were performed via DecisionLinnc 1.0 (DecisionLinnc Core Team 2023) and R software version 4.4.3.

### 2.6 Propensity Score Matching

The propensity score, the predicted probability of receiving vitamin C supplementation, was calculated by using baseline covariates in a logistic regression model. Patients were matched using the 1:4 nearest neighbor method with no replacement and a caliper width of 0.05. Subsequently, we calculated the standardized mean difference (SMD) to evaluate the effectiveness of propensity score matching (PSM) in mitigating these differences (Harder et al. 2010). In the matched cohort dataset, variables with *p* value < 0.1 in the univariable analysis were included as candidate variables in the multivariable analysis by stepwise selection. The independent variables included in the final model were in Table S4.

### 2.7 Subgroup Analyses

To determine the effect of different variables on 28‐day all‐cause mortality in patients with sepsis, we conducted a subgroup analysis within the matched cohort according to age (≥ 65 vs. < 65 years), gender (female vs. male), AKI, hypertension, diabetes, mechanical ventilation, vasopressor use, SIC score, and OASIS (≥ 36 vs. < 36).

### 2.8 Sensitivity Analysis

To verify the reliability of the findings from the matched cohort, we performed a sensitivity analysis in the unmatched cohort. Cox regression analyses were employed to identify independent prognostic factors and adjust for potential confounders. Variables with *p* value < 0.1 in the univariable analysis were integrated into the multivariable analysis to adjust for potential confounding factors. The independent variables included in the final model were in Table S5.

## 3 Result

### 3.1 Patient Characteristics

**FIGURE 1:**
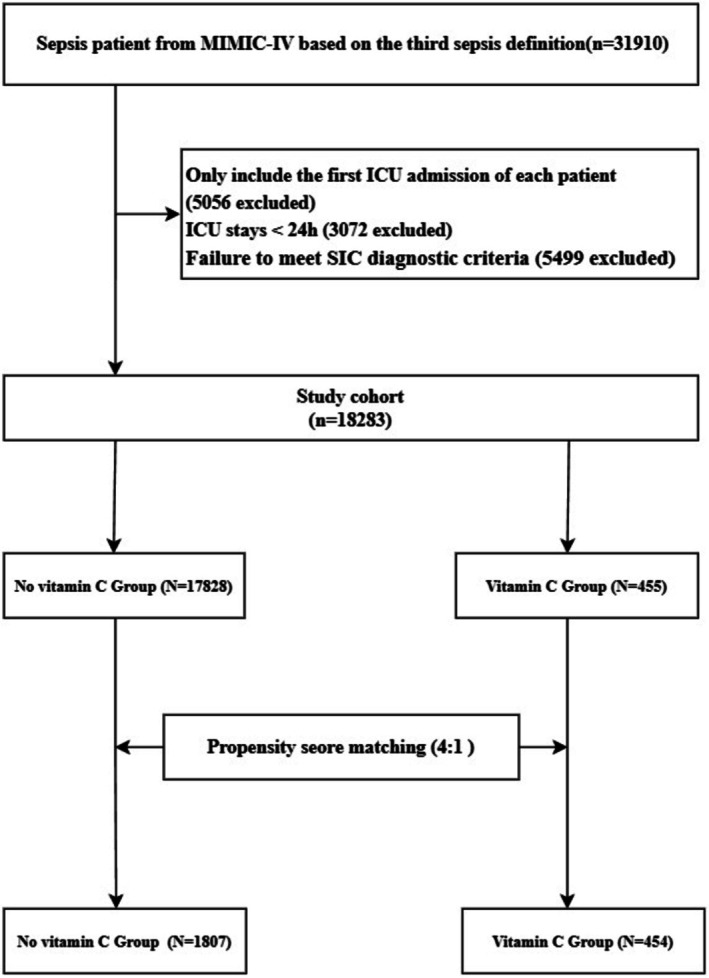
Flowchart of the study. ICU, intensive care unit; MIMIC‐IV, Medical Information‐Mart for Intensive Care IV; PSM, propensity score matching; SIC, sepsis‐induced coagulopathy.

The process of patient selection is depicted in Figure 1. A total of 31,910 patients with sepsis were identified during the study period. In the entire unmatched cohort, there were 455 cases in the vitamin C supplementation and 17,828 cases without vitamin C supplementation during their ICU stay. There were significant differences between the vitamin C and no‐vitamin C groups in terms of age, gender, disease severity scores (SOFA, APSIII, OASIS), SIC scores, comorbidities (AKI, liver cirrhosis, diabetes, CHD), and treatments (mechanical ventilation, vasopressor, heparin sodium, glucocorticosteroid); for details, see Table 1 and Table S6. After PSM, 454 patients who received vitamin C were matched with 1807 patients who did not receive vitamin C. In the matched cohort, propensity score‐matched variables were well balanced, with an SMD < 0.10, indicating that no imbalances remained for variables included in the PSM.

**TABLE 1:** Baseline characteristics in the matched cohort.

Variable names	After PSM (*N* = 2261)			
	No vitamin C (*N* = 1807)	Vitamin C (*N* = 454)	*p*	SMD
**Age (years)**	69 (58–79)	70 (58.25–79)	0.920	0.005
**Female (%)**	669 (37.02)	174 (38.33)	0.646	0.027
**Vital signs in the first 24 h**				
HR (bpm)	87 (76–104)	87 (77–103)	0.938	0.004
SBP (mmHg)	112 (99–128)	113 (99.25–128)	0.802	0.013
DBP (mmHg)	63 (54–74)	61.5 (53–72.75)	0.760	0.016
RR (bpm)	18 (15–23)	19 (14–23.75)	0.831	0.011
Temperature (°C)	36.72 (36.44–37)	36.79 (36.44–37.1)	0.746	0.015
**Laboratory tests in the first 24 h**				
pH	7.38 (7.31–7.44)	7.385 (7.3–7.43)	0.405	0.043
PaO_2_ (mm Hg)	102 (53–244.5)	95.5 (49–244.25)	0.621	0.025
PaCO_2_ (mm Hg)	40 (35–46)	40 (36–47)	0.431	0.041
Anion gap (mmol/L)	14 (11–17)	14 (11–17)	0.886	0.008
Lactate (mmol/L)	1.9 (1.4–2.9)	2 (1.4–3)	0.668	0.023
Hemoglobin (g/dL)	9.3 (7.8–10.8)	9.15 (7.7–10.9)	0.867	0.009
WBC (×10^9^/L)	11.2 (7.4–16.1)	11.5 (7.525–16.475)	0.930	0.005
PLT (×10^9^/L)	134 (90.5–208)	141 (96–207.75)	0.617	0.027
RDW	15.3 (13.9–17.3)	15.3 (14–17.4)	0.689	0.021
Creatinine (mg/dL)	1.2 (0.8–1.8)	1.1 (0.8–1.8)	0.793	0.014
BUN (mg/dL)	24 (15–41)	24 (16–42.75)	0.991	0.001
Calcium (mg/dL)	8.2 (7.7–8.7)	8.2 (7.7–8.7)	0.934	0.004
Potassium (mmol/L)	4.2 (3.8–4.7)	4.2 (3.7–4.7)	0.989	0.001
INR (ratio)	1.6 (1.5–2)	1.6 (1.5–2)	0.442	0.039
APTT (s)	34.1 (29.45–41.5)	33.9 (29.73–41.38)	0.914	0.006
SIC score (%)				
4	1050 (58.11)	277 (61.01)	0.442	0.068
5	434 (24.02)	106 (23.35)		
6	323 (17.87)	71 (15.64)		
**Disease severity scoring system**				
APSIII	50 (37–69)	49 (38–66)	0.683	0.022
OASIS	33 (27–39)	33 (28–39)	0.993	< 0.001
**Comorbidities, *n* (%)**				
Hypertension	530 (29.33)	136 (29.96)	0.838	0.014
AKI	990 (54.79)	243 (53.52)	0.667	0.025
Liver cirrhosis	290 (16.05)	69 (15.20)	0.710	0.023
Cancer	277 (15.33)	68 (14.98)	0.910	0.010
Diabetes	577 (31.93)	149 (32.82)	0.760	0.019
CHD	764 (42.28)	191 (42.07)	0.978	0.004
**Interventions in the first 24 h, *n* (%)**				
Mechanical ventilation	1556 (86.11)	394 (86.78)	0.767	0.020
Heparin sodium	1378 (76.26)	346 (76.21)	1.000	0.001
Glucocorticosteroid	568 (31.43)	146 (32.16)	0.810	0.016
Vasopressor use	1362 (75.37)	344 (75.77)	0.909	0.009

Abbreviations: AKI, acute kidney injury; APSIII, acute physiology score III; APTT, activated partial thromboplastin time; BUN, blood urea nitrogen; CHD, coronary heart disease; DBP, diastolic blood pressure; HR, heart rate; INR, international normalized ratio; OASIS, oxford acute severity of illness score; pCO_2_, partial pressure of carbon dioxide; PLT, platelet count; pO_2_, partial pressure of oxygen; PSM, propensity score matching; RDW, red blood cell width; RR, respiratory rate; SBP, systolic blood pressure; SIC, sepsis‐induced coagulopathy; SMD, standardized mean difference; WBC, white blood cell count.

### 3.2 Association Between Vitamin C and Primary and Secondary Outcomes

**FIGURE 2:**
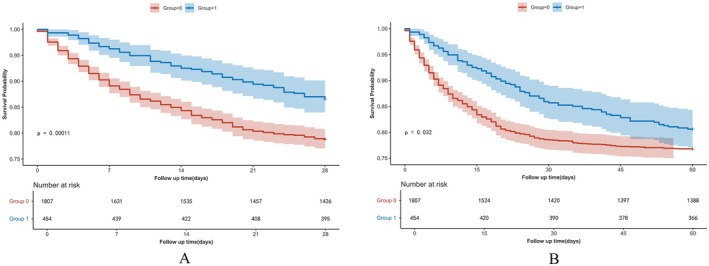
Kaplan–Meier survival curves between the two groups showing the 28‐day (A) and 60‐day (B) risk of death in patients with SIC in the matched cohort. Vitamin C users are represented by the blue line, and non‐Vitamin C users are represented by the red line.

Figure 2A displays the Kaplan–Meier curve for 28‐day all‐cause mortality stratified by vitamin C supplementation in the matched cohort. Cox regression analysis indicated that vitamin C supplementation was associated with reduced 28‐day all‐cause mortality in both univariable analysis (HR, 0.59; 95% CI, 0.45–0.77) and multivariable analysis (HR, 0.52; 95% CI, 0.40–0.69) in the matched cohort. Figure 2B displays the Kaplan–Meier curve for 60‐day all‐cause mortality stratified by vitamin C supplementation in the matched cohort. Vitamin C supplementation was associated with a lower 60‐day all‐cause mortality rate in both univariable analysis (HR, 0.78; 95% CI, 0.62–0.98) and multivariable analysis (HR, 0.69; 95% CI, 0.54–0.86) (Table 2).

**TABLE 2:** The association of vitamin C supplementation with outcomes in the matched cohort.

Outcomes	No vitamin C (*N* = 1807)	Vitamin C (*N* = 454)	Univariable analysis		Multivariable analysis^a^	
			HR/OR/MD (95% CI)	*p*	HR/OR/MD (95% CI)	*p*
**Primary outcome**						
28‐day all‐cause mortality^b^, *n* (%)	384 (21.25)	61 (13.44)	0.59 (0.45–0.77)	< 0.001	0.52 (0.40–0.69)	< 0.001
**Secondary outcomes**						
60‐day hospital mortality^b^, *n* (%)	420 (23.24)	88 (19.38)	0.78 (0.62–0.98)	0.032	0.69 (0.54–0.86)	0.001
In‐ICU mortality^c^, *n* (%)	383 (21.20)	81 (17.84)	0.81 (0.62–1.05)	0.114	0.70 (0.50–0.97)	0.035
The length of ICU stays^d^ (days), median [IQR]	3.05 [1.75–5.73]	3.92 [1.91–9.25]	0.55 (0.27–0.83)	< 0.001	0.80 (0.17–1.20)	0.003
The length of hospital stays^d^ (days), median [IQR]	9.97 [6.14–17.81]	13.60 [7.09–26.67]	2.72 (1.81–3.70)	< 0.001	3.49 (1.42–4.81)	< 0.001
Mechanical ventilation duration^d^ (h), median [IQR]	42 [21.77–90.07]	53.54 [24.51–130.11]	6.77 (1.87–11.89)	< 0.001	9.43 (0.26–15.83)	0.011

Abbreviations: CI, confidence interval; HR, hazard ratio; IQR, interquartile range; MD, median difference; OR, odds ratio.

^a^ Adjusted for age, gender, RR, DBP, HR, WBC, RDW, pCO_2_, pO_2_, pH, lactate, anion gap, creatinine, BUN, potassium, APTT, INR, SIC score, AKI, liver cirrhosis, cancer, CHD, hypertension, diabetes, OASIS, APSIII, heparin sodium, glucocorticosteroid, mechanical ventilation, and vasopressor use.

^b^ HR with 95% CI was calculated using Cox proportional hazards model.

^c^ OR with 95% CI was calculated using logistic regression model.

^d^ MD with 95% CI was calculated using Hodges–Lehmann estimator.

Logistic regression showed that vitamin C supplementation was associated with a lower ICU mortality rate in both univariable analysis (OR, 0.81; 95% CI, 0.62–1.05) and multivariable analysis (OR, 0.70; 95% CI, 0.50–0.97). In the adjusted analyses, vitamin C supplementation was also associated with a longer length of ICU stay (median difference [MD], 0.80 days; 95% CI, 0.17–1.20; *p* = 0.003) and hospital stay (MD, 3.49 days; 95% CI, 1.42–4.81; *p* < 0.001). The duration of mechanical ventilation was significantly longer in the vitamin C group (MD, 9.43 h; 95% CI, 0.26–15.83; *p* = 0.011) (Table 2).

### 3.3 Subgroup Analysis

Subgroup analyses revealed a consistent benefit of vitamin C supplementation on 28‐ and 60‐day mortality across all predefined patient characteristics. There was no statistically significant evidence of effect modification by any of the tested variables (all interaction *p* values > 0.05; for detailed forest plots, see Figure S1).

### 3.4 Effect of Dose and Duration of Vitamin C

The median duration of vitamin C supplementation was 5.19 days (IQR, 2.33–9.96). When comparing different duration ranges, it was found that short‐term (< 2 days) supplementation of vitamin C had no significant impact on survival outcomes. Continuous administration of vitamin C for exceeding 6 days was associated with reduced 28‐day all‐cause mortality risk (Table 3). Compared with the non‐vitamin C group, vitamin C supplementation at doses either ≤ 500 mg/day (median daily dose, 375 mg; IQR, 250–500 mg) or > 500 mg/day (median daily dose, 1250 mg; IQR, 750–2000 mg) significantly reduced the risk of 28‐day all‐cause mortality (Table 3).

**TABLE 3:** Duration–response and dose–response relationships between vitamin C administration and outcomes in the matched cohort.

Categories	28‐Day mortality		
	*N* (%)^b^	Crude HR (95% CI, *p*)	Adj. HR (95% CI, *p*)
**Vitamin C dose (mg/day)** ^a^			
Without vitamin C	384 (19.68%)	1	1
≤ 500	55 (14.91%)	0.66 (0.50–0.87), *p* = 0.004	0.56 (0.42–0.74), *p* < 0.001
> 500	6 (7.06%)	0.31 (0.14–0.68), *p* = 0.004	0.34 (0.15–0.76), *p* = 0.009
**Vitamin C duration (days)**			
Without vitamin C	384 (21.25%)	1	1
< 2	20 (20.20%)	0.93 (0.59–1.46), *p* = 0.755	0.79 (0.50–1.24), *p* = 0.309
2–6	18 (12.16%)	0.54 (0.33–0.86), *p* = 0.009	0.75 (0.46–1.22), *p* = 0.244
> 6	23 (11.11%)	0.48 (0.31–0.73), *p* < 0.001	0.34 (0.22–0.52), *p* < 0.001

*Note:* Cox regression was used to estimate the impact of vitamin C supplementation on mortality outcomes, adjusting for confounding variables including age, gender, RR, DBP, HR, WBC, RDW, pCO_2_, pO_2_, pH, lactate, anion gap, creatinine, BUN, potassium, APTT, INR, SIC score, AKI, liver cirrhosis, cancer, CHD, hypertension, diabetes, OASIS, APSIII, heparin sodium, glucocorticosteroid, mechanical ventilation, and vasopressor use.

Abbreviations: CI, confidence interval; HR, hazard ratio; OR, odds ratio.

^a^ The median daily dose and IQR for the ≤ 500 and > 500 mg/day subgroups were 375 mg (250–500 mg) and 1250 mg (750–2000 mg), respectively.

^b^ *N* indicates the number of death events in each category. The percentage is the mortality rate (*N*/total patients in that subgroup) × 100%.

### 3.5 Sensitivity Analyses

Figure S2 shows the Kaplan–Meier curve for 28‐ and 60‐day all‐cause mortality stratified by vitamin C supplementation in the unmatched cohort. After adjusting for potential confounders, multivariable analysis indicated that vitamin C supplementation was significantly associated with reduced 28‐day all‐cause mortality (HR, 0.58; 95% CI, 0.45–0.75) (Table S7).

## 4 Discussion

In this retrospective cohort study, our findings demonstrate that vitamin C supplementation in the ICU is associated with reduced 28‐ and 60‐day in‐hospital mortality, as well as ICU mortality among critically ill patients with SIC. However, this potential survival benefit was accompanied by longer ICU and hospital lengths of stay, as well as a longer duration of mechanical ventilation. The interpretation of these seemingly conflicting findings requires careful consideration. The prolonged supportive care durations could be a reflection of the very survival benefit observed; that is, vitamin C may have facilitated the survival of sicker patients who consequently required extended periods of organ support and in‐hospital care. Alternatively, these associations might not represent a causal harmful effect of vitamin C but could be influenced by residual confounding or random variation, particularly given the wide confidence intervals for some of these outcomes. It is also plausible that vitamin C exerts a complex biological effect that simultaneously improves survival in a subset of patients while modestly prolonging the recovery process in others. The relationship between vitamin C, survival, and resource utilization in SIC patients warrants further investigation.

Before this study, there were no studies on the correlation between vitamin C and coagulation abnormalities in patients with sepsis. Our study is the first to suggest that vitamin C may improve the prognosis of patients with SIC. The potential biological mechanisms by which vitamin C might confer benefits in SIC are multifaceted and may involve its potent antioxidant, anti‐inflammatory, and endothelial‐protective properties (Frei et al. 1989; Freedman 2008). As a key antioxidant, vitamin C can scavenge reactive oxygen species (ROS), potentially mitigating oxidative stress‐induced platelet activation and endothelial damage, thereby disrupting the vicious cycle of inflammation and coagulation (Iba et al. 2024; Levi and van der Poll 2010). Furthermore, vitamin C may modulate coagulation pathways by inhibiting pathological platelet aggregation, reducing endothelial adhesion, and enhancing fibrinolysis (Fisher et al. 2012). It also contributes to stabilizing the endothelial barrier and improving microvascular function (Oudemans‐van Straaten et al. 2014; Berger and Oudemans‐van Straaten 2015). While these mechanisms provide a plausible biological rationale, the exact pathways in SIC remain incompletely elucidated and require further investigation.

Critically ill patients frequently develop vitamin C deficiency due to increased metabolic demand and reduced intake. Supplementation with appropriate doses may reduce organ failure, decrease vasopressor requirements, accelerate recovery from organ dysfunction, and potentially lower mortality (de Spoelstra‐Man et al. 2018). An early double‐blind, placebo‐controlled trial demonstrated that aggressive supplementation with plasma ascorbate in patients with severe sepsis reduced the severity of multiorgan failure (as measured by SOFA score) and decreased levels of circulating injury biomarkers (such as C‐reactive protein and procalcitonin) (Fowler et al. 2014). A meta‐analysis indicated a trend toward reduced mortality with vitamin C, noting that monotherapy was associated with a significant reduction in mortality without increasing adverse events (Patel et al. 2022). Furthermore, a placebo‐controlled multicenter clinical trial involving 167 patients with sepsis and acute respiratory distress syndrome reported a 28‐day all‐cause mortality of 29.8% in the vitamin C group versus 46.3% in the placebo group, indicating a significant reduction in mortality with vitamin C treatment (Fowler et al. 2019).

A study investigating the duration of vitamin C supplementation found that patients receiving treatment for ≥ 5 days had a significantly lower hospital and 90‐day mortality compared to those treated for 1–2 or 3–4 days (Jung et al. 2022). This aligns with our findings, where a vitamin C duration exceeding 6 days was significantly associated with improved outcomes in SIC patients. Similarly, a secondary analysis of the LOVIT trial demonstrated no significant difference in mortality during the 4‐day vitamin C administration period. However, a substantial immediate but transient increase in mortality was observed in the vitamin C group after treatment cessation (Hemilä and Chalker 2023). Given this rebound phenomenon, our study explored mid‐term outcomes and suggests a potential 60‐day survival benefit with vitamin C in SIC patients; however, these findings warrant cautious interpretation due to the persistent treatment effect and observational nature of our study.

This study has some limitations. First, as a retrospective study employing rigorous statistical methods including PSM and multivariate analysis, our findings remain susceptible to residual bias and unmeasured confounding. Second, the single‐center design and predominantly Caucasian cohort may limit the generalizability of our findings. Third, the extended observational period introduced potential bias due to substantial improvements in SIC management protocols over time. Fourth, the lack of safety assessment of the correlation between vitamin C plasma levels and organ protection necessitates further studies to optimize clinical benefits. Finally, although we observed an association between vitamin C and reduced 60‐day mortality, this finding should be interpreted with caution. As a retrospective analysis, post‐discharge mortality may be influenced by factors unrelated to the initial sepsis event or vitamin C intervention. Therefore, the association between vitamin C and medium‐term survival requires further validation in prospective studies.

## 5 Conclusion

In conclusion, vitamin C supplementation was associated with reduced 28‐ and 60‐day all‐cause mortality, as well as ICU mortality in critically ill patients with SIC. Prospective studies are required to validate these retrospective findings.

## Author Contributions

**Ji Li:** formal analysis (equal), methodology (equal), software (equal), visualization (equal), writing – original draft (equal), writing – review and editing (equal). **Fulin Li:** data curation (equal), investigation (equal). **Dan Luo:** data curation (equal), investigation (equal). **Enling Liu:** investigation (equal), resources (equal). **Haibing Lan:** conceptualization (equal), project administration (equal), supervision (equal), writing – review and editing (equal).

## Funding

The authors have nothing to report.

## Ethics Statement

One author (J.L.), who passed the “Protecting Human Research Participants” examination, could access the database and was responsible for data extraction (Record ID: 58614822). The MIMIC‐IV database used in this study was approved by the Institutional Review Boards (IRBs) of Beth Israel Deaconess Medical Center and the Massachusetts Institute of Technology and was given a waiver of informed consent.

## Consent

The authors have nothing to report.

## Conflicts of Interest

The authors declare no conflicts of interest.

## Supporting information


**Appendix S1:** fsn371263‐sup‐0001‐AppendixS1.docx.

## Data Availability

The datasets presented in this study can be found in online repositories. The names of the repositories and accession number(s) can be found below: https://physionet.org/content/mimiciv/3.1/ (certification number: 58614822).
