# Supplementary material for: Vitamin C Supplementation During Intensive Care Unit Stay Is Associated With Improved Outcomes in Critically Ill Patients With Sepsis‐Induced Coagulopathy: A Cohort Study
Source: Food Sci Nutr. 2025 Nov 24;13(12):e71263. doi: 10.1002/fsn3.71263 (PMC12641444; doi:10.1002/fsn3.71263)
Supplement: Supplementary file 1 — Appendix S1: fsn371263‐sup‐0001‐AppendixS1.docx. [file FSN3-13-e71263-s001.docx]

***Supplementary materials***

**Table S1.** Diagnosis criteria of sepsis-induced coagulopathy

|  | **points** | **items** |
| --- | --- | --- |
| **Platelet count (10^9^/L)** | 2 | < 100 |
|  | 1 | ≥ 100, <150 |
| **INR** | 2 | > 1.4 |
|  | 1 | >1.2, ≤1.4 |
| **Total SOFA score** | 2 | ≥ 2 |
|  | 1 | 1 |

**Abbreviations:** INR, international normalized ratio.

Note: total SIC score is 4 or more with sum of SOFA score and coagulation criteria exceeding 2. Total SOFA score is the sum of four items(respiratory SOFA, cardiovascular SOFA, hepatic SOFA, and renal SOFA).

**Table S2.** Missing number (%) for included variables in the MIMIC datasets

| **Variables** | **Missing(%)** |
| --- | --- |
| Albumin | 44.4 |
| BMI | 33.6 |
| ALT | 33.3 |
| Tbil | 33.0 |
| AST | 32.5 |
| pCO2 | 18.0 |
| pO2 | 17.9 |
| PH | 16.2 |
| lactate | 16.0 |
| ventilation hour | 14.7 |
| Calcium | 4.2 |
| Temperature | 1.9 |
| SBP | 1.3 |
| DBP | 1.3 |
| APTT | 0.5 |
| Hematocrit | 0.1 |
| RDW | 0.1 |
| WBC | 0.1 |
| Creatinine | 0.1 |
| BUN | 0.1 |
| Potassium | 0.1 |
| HR | 0.0 |
| RR | 0.0 |
| Age | 0.0 |
| Hemoglobin | 0.0 |
| RBC | 0.0 |
| Anion gap | 0.0 |
| APSIII | 0.0 |
| OASIS | 0.0 |
| INR | 0.0 |
| PLT | 0.0 |
| gender | 0.0 |
| Hypertension | 0.0 |
| AKI | 0.0 |
| Liver cirrhosis | 0.0 |
| Cancer | 0.0 |
| Diabetes | 0.0 |
| CHD | 0.0 |
| Mechanical ventilation | 0.0 |
| Heparin sodium | 0.0 |
| Glucocorticosteroid | 0.0 |
| Vasopressor use | 0.0 |
| SIC score | 0.0 |

**Table S3A.** Variance inflation factor of each variable in the matched cohort.

| **Variables** | **Variance inflation factor (VIF)** |
| --- | --- |
| HR | 1.41 |
| SBP | 1.75 |
| DBP | 1.80 |
| RR | 1.25 |
| Temperature | 1.08 |
| Age | 1.55 |
| Gender | 1.11 |
| lactate | 2.14 |
| pCO2 | 1.67 |
| PH | 1.89 |
| pO2 | 1.31 |
| Hemoglobin | 1.36 |
| RDW | 1.41 |
| WBC | 1.19 |
| Creatinine | 2.06 |
| BUN | 2.17 |
| APTT | 1.22 |
| Anion gap | 2.46 |
| Calcium | 1.16 |
| Potassium | 1.27 |
| Hypertension | 1.08 |
| AKI | 1.37 |
| Liver cirrhosis | 1.60 |
| Cancer | 1.15 |
| diabetes | 1.11 |
| CHD | 1.23 |
| APSIII | 2.90 |
| oasis | 2.54 |
| Mechanical ventilation | 1.14 |
| heparin sodium | 1.14 |
| Glucocorticosteroid | 1.13 |
| Vasopressor use | 1.16 |
| Group | 1.03 |
| INR | 1.25 |
| SIC score | 2.10 |
| PLT | 1.76 |

**Table S3B.** Variance inflation factor of each variable in the unmatched cohort.

| **Variables** | **Variance inflation factor (VIF)** |
| --- | --- |
| HR | 1.34 |
| SBP | 1.11 |
| DBP | 1.02 |
| RR | 1.24 |
| Temperature | 1.03 |
| Age | 1.46 |
| Gender | 1.11 |
| lactate | 1.85 |
| pCO2 | 1.46 |
| PH | 1.73 |
| pO2 | 1.37 |
| Hemoglobin | 1.28 |
| RDW | 1.45 |
| WBC | 1.07 |
| Creatinine | 2.06 |
| BUN | 2.14 |
| APTT | 1.09 |
| Anion gap | 2.22 |
| Calcium | 1.16 |
| Potassium | 1.19 |
| Hypertension | 1.07 |
| AKI | 1.38 |
| Liver cirrhosis | 1.46 |
| Cancer | 1.09 |
| diabetes | 1.06 |
| CHD | 1.21 |
| APSIII | 2.90 |
| oasis | 2.54 |
| Mechanical ventilation | 1.10 |
| Heparin sodium | 1.16 |
| Glucocorticosteroid | 1.11 |
| Vasopressor use | 1.27 |
| Group | 1.01 |
| INR | 1.13 |
| SIC score | 1.95 |
| PLT | 1.77 |

**Table S4.** Cox regression model for 28-day all-cause mortality using stepwise selection in the matched cohort.

| Characteristics | Number(%) | HR (95%CI),*P* |
| --- | --- | --- |
| **Age** | **67.25 (15.36)** | **1.01(1.01-1.02),p<0.001** |
| **Gender** |  |  |
| **Female** | **6753 (36.9)** |  |
| **Male** | **11530 (63.1)** | **0.77(0.77-0.83),p<0.001** |
| **Temperature** | **36.65 (2.06)** | **0.98(0.98-0.99),p=0.002** |
| **RR** | **18.59 (6.68)** | **1.03(1.03-1.03),p<0.001** |
| **SBP** | **116.45 (22.77)** | **1.00(1.00-1.00),p<0.001** |
| DBP | 66.55 (65.42) | 1.00(1.00-1.00),p=0.125 |
| **HR** | **89.19 (20.25)** | **1.02(1.02-1.02),p<0.001** |
| **WBC** | **12.60 (11.84)** | **1.01(1.01-1.01),p<0.001** |
| **RDW** | **15.34 (2.61)** | **1.18(1.18-1.19),p<0.001** |
| **Hemoglobin** | **9.87 (2.15)** | **1.03(1.03-1.05),p<0.001** |
| **PLT** | **154.08 (100.61)** | **1.00(1.00-1.00),p=0.005** |
| **pCO2** | **41.08 (9.87)** | **1.01(1.01-1.01),p<0.001** |
| **pO2** | **173.94 (134.42)** | **1.00(1.00-1.00),p<0.001** |
| **pH** | **7.36 (0.11)** | **0.04(0.04-0.05),p<0.001** |
| **Lactate** | **2.80 (2.60)** | **1.12(1.12-1.13),p<0.001** |
| **Anion gap** | **13.93 (4.86)** | **1.11(1.11-1.12),p<0.001** |
| **Creatinine** | **1.49 (1.48)** | **1.16(1.16-1.18),p<0.001** |
| **BUN** | **28.17 (23.33)** | **1.02(1.02-1.02),p<0.001** |
| **Calcium** | **8.22 (0.89)** | **0.93(0.93-0.97),p=0.001** |
| **Potassium** | **4.24 (0.74)** | **1.26(1.26-1.32),p<0.001** |
| **PTT** | **40.56 (24.00)** | **1.01(1.01-1.01),p<0.001** |
| **INR** | **1.87 (1.06)** | **1.16(1.14,1.18),p<0.001** |
| **SIC score** |  |  |
| **4** | **10479 (57.3)** |  |
| **5** | **4893 (26.8)** | **0.95(0.95-1.04),p=0.265** |
| **6** | **2911 (15.9)** | **1.80(1.80-1.98),p<0.001** |
| **AKI** |  |  |
| **No** | **10921 (59.7)** |  |
| **Yes** | **7362 (40.3)** | **3.91(3.91-4.25),p<0.001** |
| **Liver cirrhosis** |  |  |
| **No** | **15777 (86.3)** |  |
| **Yes** | **2506 (13.7)** | **2.17(2.17-2.37),p<0.001** |
| **Cancer** |  |  |
| **No** | **15166 (83.0)** |  |
| **Yes** | **3117 (17.0)** | **1.44(1.44-1.58).p<0.001** |
| **CHD** |  |  |
| **No** | **10330 (56.5)** |  |
| **Yes** | **7953 (43.5)** | **0.69(0.69-0.74),p<0.001** |
| **Hypertension** |  |  |
| **No** | **10707 (58.6)** |  |
| **Yes** | **7576 (41.4)** | **0.67(0.67-0.72),p<0.001** |
| Diabetes |  |  |
| No | 12836 (70.2) |  |
| Yes | 5447 (29.8) | 0.94(0.94-1.02),p=0.135 |
| **OASIS** | **32.27 (8.62)** | **1.10(1.10-1.10),p<0.001** |
| **APSIII** | **49.46 (22.61)** | **1.04(1.04-1.04),p<0.001** |
| **Heparin sodium** |  |  |
| **No** | **6359 (34.8)** |  |
| **Yes** | **11924 (65.2)** | **1.80(1.80-1.97),p<0.001** |
|  |  |  |
| **No** | **14095 (77.1)** |  |
| **Yes** | **4188 (22.9)** | **2.01(2.01-2.18),p<0.001** |
| **Mechanical ventilation** |  |  |
| **No** | **2680 (14.7)** |  |
| **Yes** | **15603 (85.3)** | **1.18(1.18-1.32),p=0.006** |
| **Vasopressor use** |  |  |
| **No** | **6222 (34.0)** |  |
| **Yes** | **12061 (66.0)** | **2.04(2.04-2.24),p<0.001** |
| **Vitamin C** |  |  |
| **No** | **17828 (97.5)** |  |
| **Yes** | **455 ( 2.5)** | **0.90(0.90-1.16),p=0.399** |

**Table S5.**Cox regression model for 28-day all-cause mortality using stepwise selection in the unmatched cohort.

| Characteristics | Number(%) | HR (95%CI),*P* |
| --- | --- | --- |
| **Age** | **67.25 (15.36)** | **1.01(1.01-1.02),p<0.001** |
| **Gender** |  |  |
| **Female** | **6753 (36.9)** |  |
| **Male** | **11530 (63.1)** | **0.77(0.77-0.83),p<0.001** |
| **Temperature** | **36.65 (2.06)** | **0.98(0.98-0.99),p=0.002** |
| **RR** | **18.59 (6.68)** | **1.03(1.03-1.03),p<0.001** |
| **SBP** | **116.45 (22.77)** | **1.00(1.00-1.00),p<0.001** |
| DBP | 66.55 (65.42) | 1.00(1.00-1.00),p=0.125 |
| **HR** | **89.19 (20.25)** | **1.02(1.02-1.02),p<0.001** |
| **WBC** | **12.60 (11.84)** | **1.01(1.01-1.01),p<0.001** |
| **RDW** | **15.34 (2.61)** | **1.18(1.18-1.19),p<0.001** |
| **Hemoglobin** | **9.87 (2.15)** | **1.03(1.03-1.05),p<0.001** |
| **PLT** | **154.08 (100.61)** | **1.00(1.00-1.00),p=0.005** |
| **PaCO2** | **41.08 (9.87)** | **1.01(1.01-1.01),p<0.001** |
| **PaO2** | **173.94 (134.42)** | **1.00(1.00-1.00),p<0.001** |
| **pH** | **7.36 (0.11)** | **0.04(0.04-0.05),p<0.001** |
| **Lactate** | **2.80 (2.60)** | **1.12(1.12-1.13),p<0.001** |
| **Anion gap** | **13.93 (4.86)** | **1.11(1.11-1.12),p<0.001** |
| **Creatinine** | **1.49 (1.48)** | **1.16(1.16-1.18),p<0.001** |
| **BUN** | **28.17 (23.33)** | **1.02(1.02-1.02),p<0.001** |
| **Calcium** | **8.22 (0.89)** | **0.93(0.93-0.97),p=0.001** |
| **Potassium** | **4.24 (0.74)** | **1.26(1.26-1.32),p<0.001** |
| **PTT** | **40.56 (24.00)** | **1.01(1.01-1.01),p<0.001** |
| **INR** | **1.87 (1.06)** | **1.16(1.14,1.18),p<0.001** |
| **SIC score** |  |  |
| **4** | **10479 (57.3)** |  |
| **5** | **4893 (26.8)** | **0.95(0.95-1.04),p=0.265** |
| **6** | **2911 (15.9)** | **1.80(1.80-1.98),p<0.001** |
| **AKI** |  |  |
| **No** | **10921 (59.7)** |  |
| **Yes** | **7362 (40.3)** | **3.91(3.91-4.25),p<0.001** |
| **Liver cirrhosis** |  |  |
| **No** | **15777 (86.3)** |  |
| **Yes** | **2506 (13.7)** | **2.17(2.17-2.37),p<0.001** |
| **Cancer** |  |  |
| **No** | **15166 (83.0)** |  |
| **Yes** | **3117 (17.0)** | **1.44(1.44-1.58).p<0.001** |
| **CHD** |  |  |
| **No** | **10330 (56.5)** |  |
| **Yes** | **7953 (43.5)** | **0.69(0.69-0.74),p<0.001** |
| **Hypertension** |  |  |
| **No** | **10707 (58.6)** |  |
| **Yes** | **7576 (41.4)** | **0.67(0.67-0.72),p<0.001** |
| Diabetes |  |  |
| No | 12836 (70.2) |  |
| Yes | 5447 (29.8) | 0.94(0.94-1.02),p=0.135 |
| **OASIS** | **32.27 (8.62)** | **1.10(1.10-1.10),p<0.001** |
| **APSIII** | **49.46 (22.61)** | **1.04(1.04-1.04),p<0.001** |
| **Heparin sodium** |  |  |
| **No** | **6359 (34.8)** |  |
| **Yes** | **11924 (65.2)** | **1.80(1.80-1.97),p<0.001** |
|  |  |  |
| **No** | **14095 (77.1)** |  |
| **Yes** | **4188 (22.9)** | **2.01(2.01-2.18),p<0.001** |
| **Mechanical ventilation** |  |  |
| **No** | **2680 (14.7)** |  |
| **Yes** | **15603 (85.3)** | **1.18(1.18-1.32),p=0.006** |
| **Vasopressor use** |  |  |
| **No** | **6222 (34.0)** |  |
| **Yes** | **12061 (66.0)** | **2.04(2.04-2.24),p<0.001** |
| **Vitamin C** |  |  |
| **No** | **17828 (97.5)** |  |
| **Yes** | **455 ( 2.5)** | **0.90(0.90-1.16),p=0.399** |

**Table S6.** Baseline characteristics in the umatched cohort.

| **Variable Names** | **Before PSM(N=18283)** | | | |
| --- | --- | --- | --- | --- |
|  | **No vitamin C (N=17828)** | **Vitamin C**  **(N=455)** | **p** | **SMD** |
| **Age (years)** | 69 (58-79) | 70 (58-79) | 0.914 | 0.005 |
| **Femal (%)** | 6578 (36.90) | 175 (38.46) | 0.526 | 0.032 |
| **Vital signs in the first 24 h** |  |  |  |  |
| HR (bpm) | 85 (76-101) | 87 (77-103) | 0.238 | 0.054 |
| SBP (mmHg) | 114 (100-130) | 113 (99-128) | 0.038 | 0.101 |
| DBP (mmHg) | 64 (54-75) | 61 (53-72.5) | 0.561 | 0.037 |
| RR (bpm) | 18 (14-22) | 19 (14-24) | 0.011 | 0.115 |
| Temperature (°C) | 36.70 (36.44-37.06) | 36.78 (36.4-37.11) | 0.366 | 0.045 |
| **Laboratory tests in the first 24 h** |  |  |  |  |
| pH | 7.39 (7.31-7.44) | 7.38 (7.3-7.43) | 0.450 | 0.036 |
| PaO2 (mm Hg) | 119 (64-290) | 95 (49-243.5) | 0.004 | 0.138 |
| PaCO2 (mm Hg) | 40 (35-46) | 40 (36-47) | 0.024 | 0.100 |
| Anion gap (mmol/L) | 13 (11-16) | 14 (11-17) | 0.015 | 0.114 |
| lactate (mmol/L) | 2 (1.4-2.9) | 2 (1.35-3) | 0.416 | 0.040 |
| Hemoglobin (g/dL) | 9.7 (8.3-11.2) | 9.2 (7.7-10.9) | <0.001 | 0.185 |
| WBC (×10^9/L) | 10.7 (7.3-15.2) | 11.5 (7.55-16.5) | 0.036 | 0.101 |
| PLT (×10^9/L) | 130 (93-185) | 141 (95.5-207.5) | 0.002 | 0.144 |
| RDW | 14.6 (13.5-16.5) | 15.3 (14-17.4) | <0.001 | 0.257 |
| Creatinine (mg/dL) | 1 (0.8-1.6) | 1.1 (0.8-1.8) | 0.004 | 0.128 |
| BUN (mg/dL) | 20 (14-34) | 24 (16-42.5) | <0.001 | 0.186 |
| Calcium (mg/dL) | 8.2 (7.8-8.7) | 8.2 (7.7-8.7) | 0.600 | 0.025 |
| Potassium (mmol/L) | 4.2 (3.8-4.6) | 4.2 (3.7-4.7) | 0.413 | 0.039 |
| INR (ratio) | 1.6 (1.4-1.9) | 1.6 (1.5-2) | 0.011 | 0.108 |
| APTT (sec) | 33.3 (29-40.8) | 33.9 (29.7-41.35) | 0.609 | 0.025 |
| SIC score (%) |  |  |  |  |
| 4 | 10201 (57.22) | 278 (61.10) | 0.193 | 0.088 |
| 5 | 4787 (26.85) | 106 (23.30) |  |  |
| 6 | 2840 (15.93) | 71 (15.60) |  |  |
| **Disease severity scoring system** |  |  |  |  |
| APSIII | 44 (33-61) | 49 (38-66) | <0.001 | 0.195 |
| OASIS | 32 (26-38) | 33 (28-39) | 0.002 | 0.145 |
| **Comorbidities, n(%)** |  |  |  |  |
| Hypertension | 7440 (41.73) | 136 (29.89) | <0.001 | 0.249 |
| AKI | 7118 (39.93) | 244 (53.63) | <0.001 | 0.277 |
| Liver cirrhosis | 2437 (13.67) | 69 (15.16) | 0.397 | 0.043 |
| Cancer | 3049 (17.10) | 68 (14.95) | 0.252 | 0.059 |
| Diabetes | 5298 (29.72) | 149 (32.75) | 0.179 | 0.065 |
| CHD | 7762 (43.54) | 191 (41.98) | 0.539 | 0.032 |
| **Interventions in the first 24h, n(%)** |  |  |  |  |
| Mechanical ventilation | 15208 (85.30) | 395 (86.81) | 0.406 | 0.044 |
| Heparin sodium | 11577 (64.94) | 347 (76.26) | <0.001 | 0.251 |
| Glucocorticosteroid | 4041 (22.67) | 147 (32.31) | <0.001 | 0.217 |
| Vasopressor use | 11716 (65.72) | 345 (75.82) | <0.001 | 0.224 |

Abbreviations: PSM: propensity score matching; SMD: standardized mean difference, HR: heart rate ; SBP: systolic blood pressure; DBP: diastolic blood pressure; RR: respiratory rate; pO2: partial pressure of oxygen; pCO2: partial assure of carbon dioxide; WBC: white blood cell count; PLT: Platelet count; RDW: red blood cell width; BUN: blood urea nitrogen, INR: international normalized ratio; APTT: activated partial thromboplastin time, SIC: sepsis-induced coagulopathy; APSIII: acute physiology score III; OASIS: oxford acute severity of illness score; AKI: acute kidney injury; CHD: coronary heart disease.

**Table S7A.** 28-day mortality Univariable analysis and Multivariable analysis in the matched cohort

| Characteristics | Number(%) | HR (univariable) | HR(multivariable) |
| --- | --- | --- | --- |
| Age | 67.34 (15.69) | 1.01(1.00-1.02),p=0.001 | 1.02(1.01-1.02),p<0.001 |
| Gender |  |  |  |
| Female | 843 (37.3) |  |  |
| Male | 1418 (62.7) | 0.90(0.75-1.09),p=0.299 | 1.13(0.93-1.38),p=0.217 |
| Temperature | 36.71 (1.44) | 1.00(0.94-1.07),p=0.953 |  |
| RR | 19.40 (6.95) | 1.05(1.03-1.06),p<0.001 | 1.01(1.00-1.03),p=0.103 |
| SBP | 114.54 (22.22) | 1.00(0.99-1.00),p=0.103 |  |
| DBP | 65.06 (17.32) | 1.00(1.00-1.01),p=0.111 | 1.00(1.00-1.01),p=0.179 |
| HR | 90.42 (20.89) | 1.02(1.01-1.02),p<0.001 | 1.00(1.00-1.01),p=0.073 |
| WBC | 13.79 (16.08) | 1.00(1.00-1.01),p=0.039 | 1.00(0.99-1.00),p=0.280 |
| RDW | 15.99 (2.90) | 1.16(1.13-1.19),p<0.001 | 1.09(1.06-1.12),p<0.001 |
| Hemoglobin | 9.46 (2.12) | 1.03(0.99-1.07),p=0.205 |  |
| PLT | 166.53 (118.93) | 1.00(1.00-1.00),p=0.734 |  |
| pCO2 | 41.65 (11.16) | 1.01(1.01-1.02),p<0.001 | 1.01(1.00-1.02),p=0.003 |
| pO2 | 153.43 (127.71) | 1.00(0.99-1.00),p<0.001 | 1.00(1.00-1.00),p<0.001 |
| pH | 7.36 (0.11) | 0.05(0.02-0.10),p<0.001 | 0.94(0.33-2.69),p=0.908 |
| Lactate | 2.66 (2.41) | 1.13(1.10-1.16),p<0.001 | 1.03(0.99-1.07),p=0.18 |
| Anion gap | 14.54 (5.06) | 1.09(1.07-1.10),p<0.001 | 1.03(1.01-1.06),p=0.005 |
| Creatinine | 1.67 (1.60) | 1.10(1.06-1.15),p<0.001 | 0.92(0.84-1.01),p=0.067 |
| BUN | 32.62 (25.91) | 1.01(1.01-1.01),p<0.001 | 1.00(1.00-1.01),p=0.932 |
| Calcium | 8.20 (0.93) | 1.08(0.97-1.20),p=0.144 |  |
| Potassium | 4.26 (0.77) | 1.18(1.05-1.32),p=0.006 | 0.97(0.86-1.09),p=0.593 |
| APTT | 41.06 (23.81) | 1.01(1.01-1.01),p<0.001 | 1.01(1.00-1.01),p=0.003 |
| INR | 1.96 (1.15) | 1.17(1.12-1.23),p<0.001 | 1.05(0.98-1.13),p=0.149 |
| SIC score |  |  |  |
| 4 | 1327 (58.7) |  |  |
| 5 | 540 (23.9) | 0.75(0.58-0.96),p=0.023 | 0.81(0.63-1.06),p=0.120 |
| 6 | 394 (17.4) | 1.50(1.20-1.87),p<0.001 | 0.99(0.76-1.28),p=0.993 |
| AKI |  |  |  |
| No | 1028 (45.5) |  |  |
| Yes | 1233 (54.5) | 3.35(2.68-4.20),p<0.001 | 1.38(1.06-1.79),p=0.018 |
| Liver cirrhosis |  |  |  |
| No | 1902 (84.1) |  |  |
| Yes | 359 (15.9) | 1.97(1.59-2.44),p<0.001 | 1.11(0.85-1.46),p=0.43 |
| Cancer |  |  |  |
| No | 1916 (84.7) |  |  |
| Yes | 345 (15.3) | 1.39(1.10-1.76),p=0.006 | 1.42(1.10-1.82),p=0.006 |
| CHD |  |  |  |
| No | 1306 (57.8) |  |  |
| Yes | 955 (42.2) | 0.78(0.65-0.95),p=0.012 | 1.06(0.86-1.31),p=0.606 |
| Hypertension |  |  |  |
| No | 1595 (70.5) |  |  |
| Yes | 666 (29.5) | 0.76(0.62-0.95),p=0.104 | 1.03(0.82-1.28),p=0.814 |
| Diabetes |  |  |  |
| No | 1535 (67.9) |  |  |
| Yes | 726 (32.1) | 0.75(0.61-0.93),p=0.007 | 0.72(0.58-0.89),p=0.003 |
| OASIS | 33.49 (8.91) | 1.08(1.07-1.09),p<0.001 | 1.02(1.01-1.04),p=0.007 |
| APSIII | 54.17 (23.46) | 1.03(1.03-1.03),p<0.001 | 1.02(1.01-1.02),p<0.001 |
| Heparin sodium |  |  |  |
| No | 537 (23.8) |  |  |
| Yes | 1724 (76.2) | 1.52(1.19-1.95),p=0.001 | 0.74(0.57-0.96),p=0.025 |
| Glucocorticosteroid |  |  |  |
| No | 1547 (68.4) |  |  |
| Yes | 714 (31.6) | 1.74(1.45-2.10),p<0.001 | 1.11(0.91-1.35),p=0.313 |
| Mechanical ventilation |  |  |  |
| No | 311 (13.8) |  |  |
| Yes | 1950 (86.2) | 1.05(0.80-1.39),p=0.720 | 0.82(0.61-1.10),p=0.187 |
| Vasopressor use |  |  |  |
| No | 555 (24.5) |  |  |
| Yes | 1706 (75.5) | 3.14(2.31-4.28),p<0.001 | 2.98(2.14-4.14),p<0.001 |
| Vitamin C |  |  |  |
| No | 1807 (79.9) |  |  |
| Yes | 454 (20.1) | **0.59(0.45-0.77),p<0.001** | **0.52(0.40-0.69),p<0.001** |

**Table S7B.** 28-day mortality Univariable analysis and Multivariable analysis in the unmatched cohort

| Characteristics | Number(%) | HR (univariable) | HR(multivariable) |
| --- | --- | --- | --- |
| Age | 67.25 (15.36) | 1.01(1.01-1.02),p<0.001 | 1.02(1.01-1.02),p<0.001 |
| Gender |  |  |  |
| Female | 6753 (36.9) |  |  |
| Male | 11530 (63.1) | 0.77(0.77-0.83),p<0.001 | 0.95(0.87-1.03),p=0.183 |
| Temperature | 36.65 (2.06) | 0.98(0.98-0.99),p=0.002 | 1.00(0.98-1.01),p=0.658 |
| RR | 18.59 (6.68) | 1.03(1.03-1.03),p<0.001 | 1.01(1.00-1.01),p=0.003 |
| SBP | 116.45 (22.77) | 1.00(1.00-1.00),p<0.001 | 1.00(1.00-1.00),p=0.033 |
| DBP | 66.55 (65.42) | 1.00(1.00-1.00),p=0.125 |  |
| HR | 89.19 (20.25) | 1.02(1.02-1.02),p<0.001 | 1.00(1.00-1.00),p=0.776 |
| WBC | 12.60 (11.84) | 1.01(1.01-1.01),p<0.001 | 1.00(1.00-1.01),p=0.052 |
| RDW | 15.34 (2.61) | 1.18(1.18-1.19),p<0.001 | 1.08(1.06-1.09),p<0.001 |
| Hemoglobin | 9.87 (2.15) | 1.03(1.03-1.05),p<0.001 | 1.07(1.05-1.09),p<0.001 |
| PLT | 154.08 (100.61) | 1.00(1.00-1.00),p=0.005 | 1.00(1.00-1.00),p=0.076 |
| pCO2 | 41.08 (9.87) | 1.01(1.01-1.01),p<0.001 | 1.01(1.01-1.01),p<0.001 |
| pO2 | 173.94 (134.42) | 1.00(1.00-1.00),p<0.001 | 1.00(1.00-1.00),p<0.001 |
| pH | 7.36 (0.11) | 0.04(0.04-0.05),p<0.001 | 0.78(0.52-1.16),p=0.213 |
| Lactate | 2.80 (2.60) | 1.12(1.12-1.13),p<0.001 | 1.02(1.01-1.04),p=0.003 |
| Anion gap | 13.93 (4.86) | 1.11(1.11-1.12),p<0.001 | 1.04(1.03-1.05),p<0.001 |
| Creatinine | 1.49 (1.48) | 1.16(1.16-1.18),p<0.001 | 0.93(0.90-0.97),p<0.001 |
| BUN | 28.17 (23.33) | 1.02(1.02-1.02),p<0.001 | 1.00(1.00-1.01),p=0.003 |
| Calcium | 8.22 (0.89) | 0.93(0.93-0.97),p=0.001 | 1.00(0.96-1.04),p=0.875 |
| Potassium | 4.24 (0.74) | 1.26(1.26-1.32),p<0.001 | 0.98(0.94-1.03),p=0.478 |
| PTT | 40.56 (24.00) | 1.01(1.01-1.01),p<0.001 | 1.01(1.00-1.01),p<0.001 |
| INR | 1.87 (1.06) | 1.16(1.14,1.18),p<0.001 | 1.00(0.97-1.03),p=0.969 |
| SIC score |  |  |  |
| 4 | 10479 (57.3) |  |  |
| 5 | 4893 (26.8) | 0.95(0.95-1.04),p=0.265 | 1.12(1.00-1.25),p=0.046 |
| 6 | 2911 (15.9) | 1.80(1.80-1.98),p<0.001 | 1.25(1.10-1.42),p<0.001 |
| AKI |  |  |  |
| No | 10921 (59.7) |  |  |
| Yes | 7362 (40.3) | 3.91(3.91-4.25),p<0.001 | 1.45(1.32-1.60),p<0.001 |
| Liver cirrhosis |  |  |  |
| No | 15777 (86.3) |  |  |
| Yes | 2506 (13.7) | 2.17(2.17-2.37),p<0.001 | 1.32(1.18-1.47),p<0.001 |
| Cancer |  |  |  |
| No | 15166 (83.0) |  |  |
| Yes | 3117 (17.0) | 1.44(1.44-1.58).p<0.001 | 1.36(1.23-1.49),p<0.001 |
| CHD |  |  |  |
| No | 10330 (56.5) |  |  |
| Yes | 7953 (43.5) | 0.69(0.69-0.74),p<0.001 | 0.86(0.79-0.94),p=0.001 |
| Hypertension |  |  |  |
| No | 10707 (58.6) |  |  |
| Yes | 7576 (41.4) | 0.67(0.67-0.72),p<0.001 | 0.97(0.89-1.05),p=0.442 |
| Diabetes |  |  |  |
| No | 12836 (70.2) |  |  |
| Yes | 5447 (29.8) | 0.94(0.94-1.02),p=0.135 |  |
| OASIS | 32.27 (8.62) | 1.10(1.10-1.10),p<0.001 | 1.03(1.02-1.04),p<0.001 |
| APSIII | 49.46 (22.61) | 1.04(1.04-1.04),p<0.001 | 1.02(1.01-1.02),p<0.001 |
| Heparin sodium |  |  |  |
| No | 6359 (34.8) |  |  |
| Yes | 11924 (65.2) | 1.80(1.80-1.97),p<0.001 | 0.78(0.71-0.86),p<0.001 |
|  |  |  |  |
| No | 14095 (77.1) |  |  |
| Yes | 4188 (22.9) | 2.01(2.01-2.18),p<0.001 | 1.15(1.06-1.25),p=0.001 |
| Mechanical ventilation |  |  |  |
| No | 2680 (14.7) |  |  |
| Yes | 15603 (85.3) | 1.18(1.18-1.32),p=0.006 | 0.93(0.82-1.04),p=0.209 |
| Vasopressor use |  |  |  |
| No | 6222 (34.0) |  |  |
| Yes | 12061 (66.0) | 2.04(2.04-2.24),p<0.001 | 1.94(1.74-2.15),p<0.001 |
| Vitamin C |  |  |  |
| No | 17828 (97.5) |  |  |
| Yes | 455 ( 2.5) | **0.90(0.90-1.16),p=0.399** | **0.58(0.45-0.75),p<0.001** |

**Figure S1.** Subgroup analyses for 28-day(A) and 60-day(B) all-cause mortality in the matched cohort.


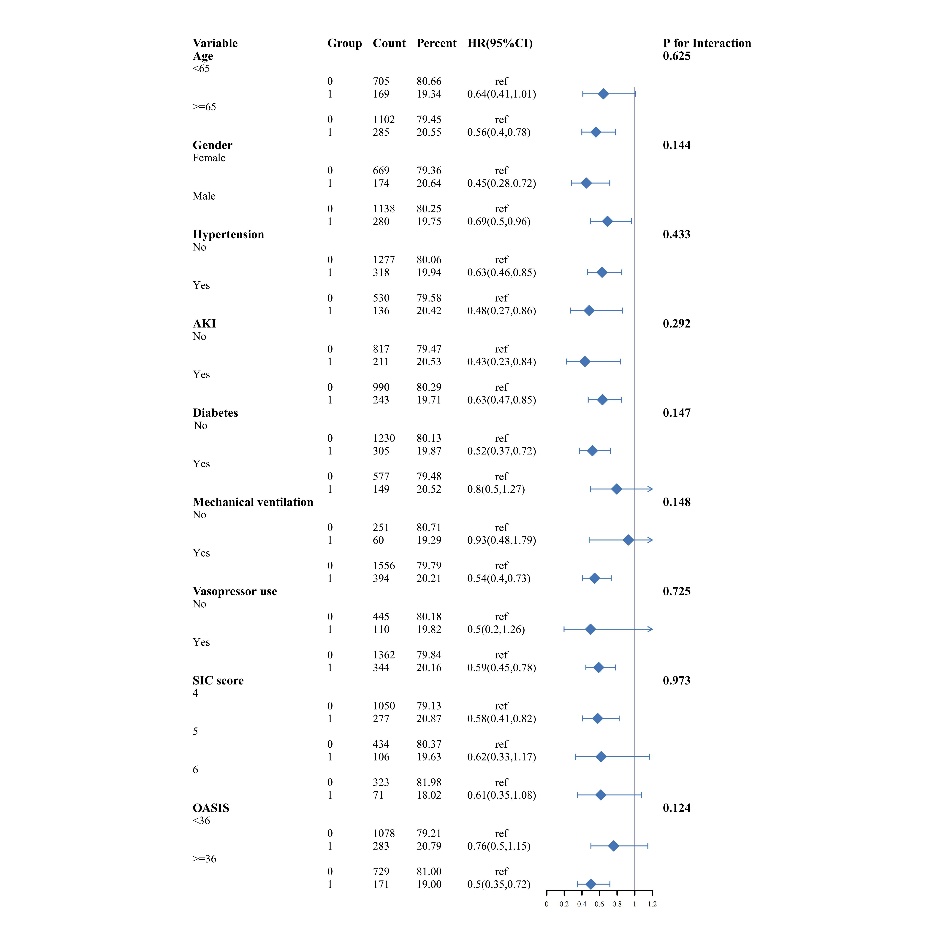

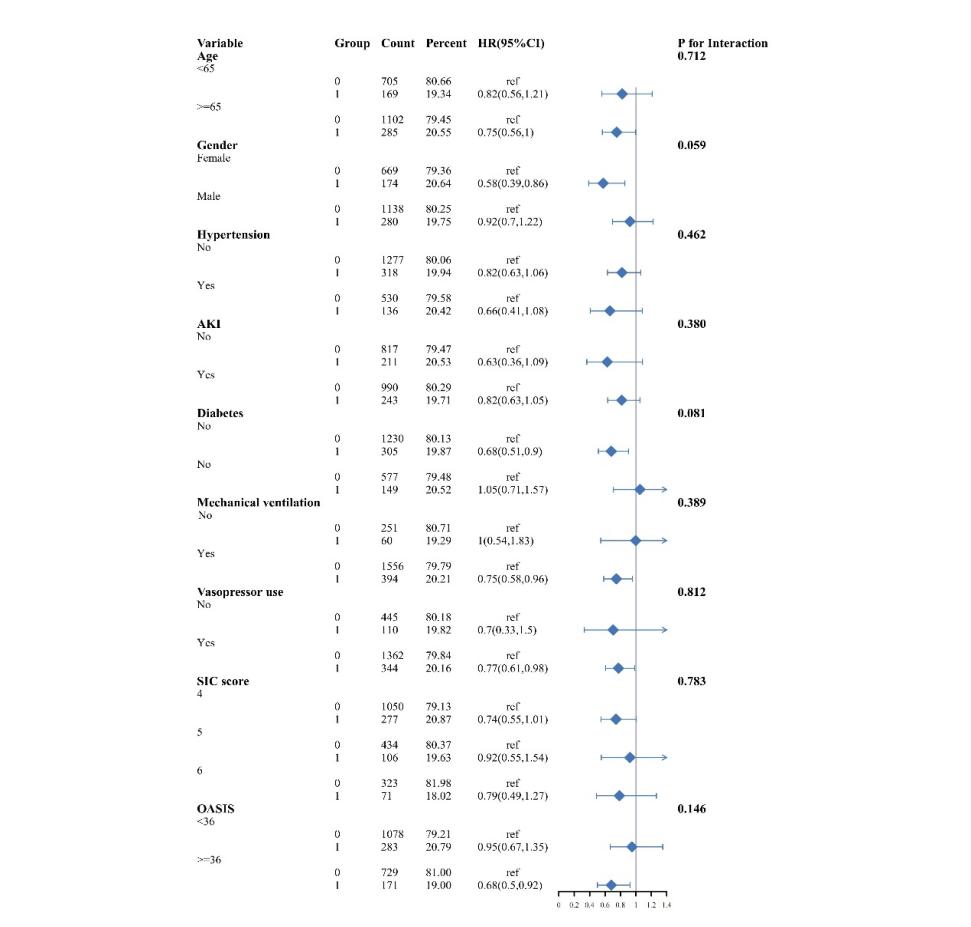


A

B

**Figure S2**. Kalpan-Meier survival curves between the two groups showing the 28-day(A) and 60-day(B) risk of death in patients with SIC in the unmatched cohort. Vitamin C users are represented by the blue line, and non-Vitamin C users are represented by the red line.


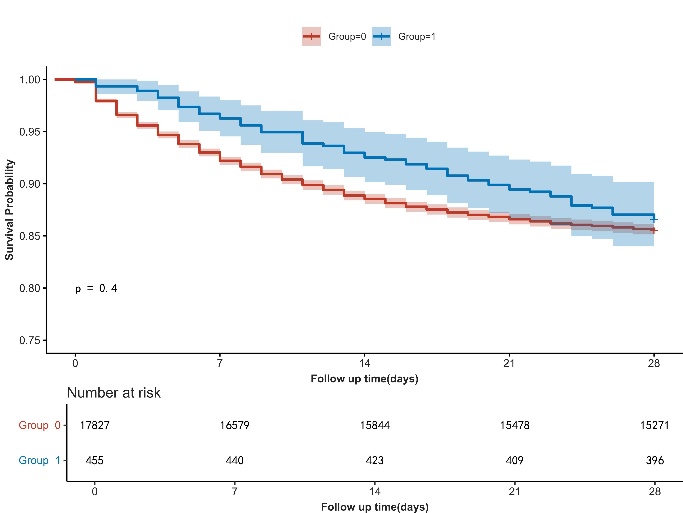

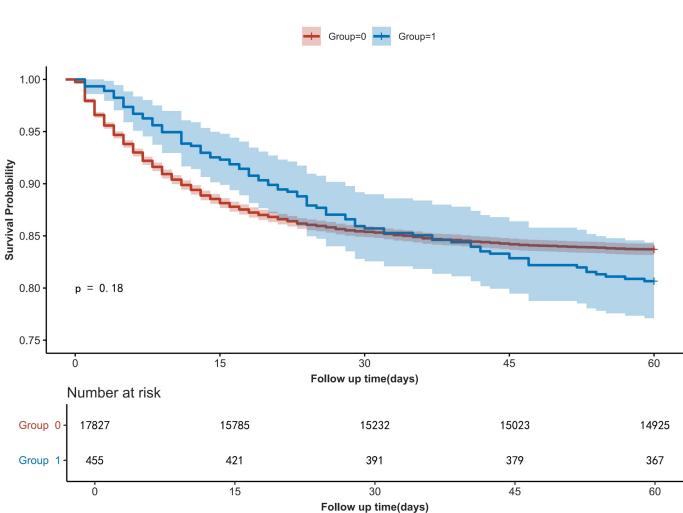


A

B
